# Fabrication and Mechanical Properties of Tungsten Inert Gas Welding Ring Welded Joint of 7A05-T6/5A06-O Dissimilar Aluminum Alloy

**DOI:** 10.3390/ma11071156

**Published:** 2018-07-06

**Authors:** Wukun Wang, Zengqiang Cao, Kai Liu, Xianglong Zhang, Kewen Zhou, Peng Ou

**Affiliations:** 1 School of Mechanical Engineering, Northwestern Polytechnical University, 127 Youyi Ave. West, Xi’an 710072, China; wwkun@mail.nwpu.edu.cn (W.W.); 2018100130@mail.nwpu.edu.cn (P.O.); 2 Kunming Precision Machinery Research Institute, P.O. Box No. 26, Kunming 650118, China; Zhangxianglong1983@163.com (X.Z.); 13759138258@139.com (K.Z.); 3 College of Naval Architecture and Ocean Engineering, Naval University of Engineering, P.O. Box No. 076, Wuhan 430033, China

**Keywords:** TIG welding, similar quenching effect, heat-affected zone, Aluminum alloys, heat input, microstructural characterization

## Abstract

In this paper, Tungsten Inert Gas Welding (TIG) and single-sided welding and double-sided forming have been used to weld the 7A05-T6/5A06-O dissimilar aluminum alloy circular welded joint of a ring-stiffened closed cylindrical sandwich shell. Microstructural characterization and mechanical properties of welded joints were investigated by use of scanning electron microscopy (SEM), backscatter electron diffraction (EBSD) and transmission electron microscopy (TEM), respectively. Hardness distribution and tensile properties of the welded joints were examined. The results showed the failure of the welded joints produced in the fusion zone (FZ). The tensile strength and yield strength of the welded joints were, respectively, 78.87% and 97.24% of the 5A06-O base metal (BM), and the elongation reached 84.29% of 7A05-T6 base metal. Welding high heat input led to the coarse grain size in the fusion zone, and the long-term similar quenching effect can lead to the full dissolution of the strengthening zone of the fusion zone resulting in the reductions of strength and hardness. Around 7A05 heat-affected zone (HAZ), there is an obvious hardening zone and softening zone, the solid solution precipitates into the Rayleigh brilliant η′ (MgZn_2_) phase, which results in natural aging strengthening, thus obtaining high hardness. η′ (MgZn_2_) enhanced phase dissolved fully and the dislocation density decreased rapidly in the HAZ region resulted in a softening zone with a lower hardness at about (10–18) mm from the center of the weld center.

## 1. Introduction

**Figure 1:**
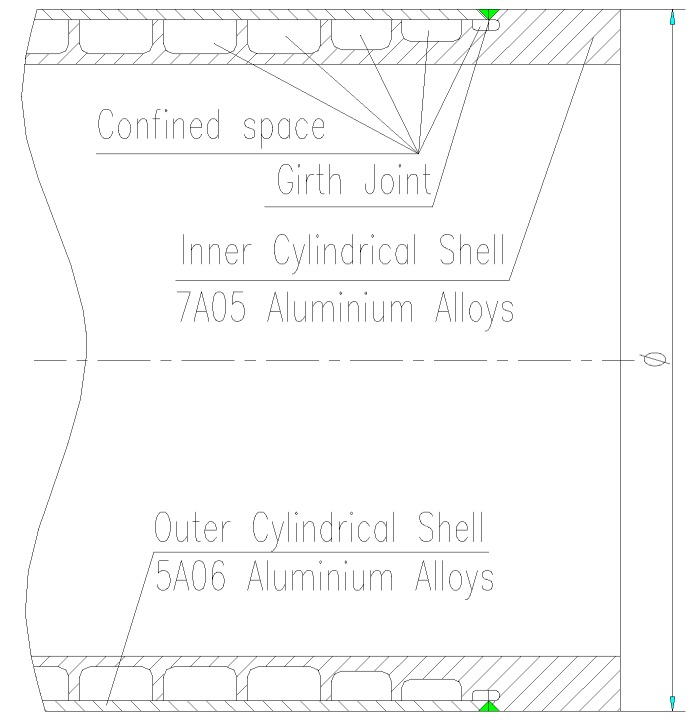
Sample structure sketch.

Sandwich cylindrical shell structures not only effectively reduce the weight of the structure, but also have excellent heat dissipation, vibration control and energy consumption characteristics [1,2,3]. Therefore, this structure has a wide application prospect in aerospace, marine, vehicle engineering and construction fields etc. Figure 1 shows a typical sandwich cylindrical shell used in the deep water. 7A05-T6 acts as the core to bear the stress delivered by the housing (5A06-O). The key technology to fabricate such structure is how to weld these two dissimilar aluminum alloys.

7A05 belongs to the 7XXX series aluminum alloy, which is a high-strength aluminum alloy that usually exhibits softening in the heat affection zone of the fusion-welded joints. More importantly, the thermal cracking and porosity in the fusion-welded joints makes this alloy difficult to weld in theory. In comparison, 5A06-O aluminum alloy is widely used as an important structural material in the marine environment for its excellent weldability and corrosion resistance, moderate strength, and excellent extensibility [4,5,6]. Numerous studies have been carried out on the welding of aluminum alloy [7,8,9]. Giri and Kainuma et al. [10,11] have considered the test method of residual stress of welded joints. Krolczyk et al. [12,13] have studied the influence of argon pollution on the weld surface morphology and different methods for measuring the weld joint area. Kumar et al. [14] have studied wear characteristics and defects analysis of friction-stir welded joint. Several pieces of research have reported the homogeneous welding of the two similar aluminum alloys [15,16]; however, the heterogeneous connection problems of the above two kinds of aluminum alloys are not discussed at great length. In particular, there are no reports on the welding of 7A05-T6/5A06-O aluminum alloy ring-welded joints with the ring-stiffened closed cylindrical sandwich shell. Besides the welding materials, the welding methods of dissimilar aluminum alloys also have a great influence on mechanical properties of welded joints, which mainly include friction-stir welding (FSW) [17,18], laser welding [19], metal inert-gas welding (MIG( and TIG) [20,21], resistance welding [22]. TIG welding is the preferred method for thin-walled aluminum alloys due to the ease of handling and the admirable economy [23], which is prone to produce high quality and defect-free welded joints [24]. Despite the 36advantages of TIG, there continue to be many problems in the welding such as porosity, liquation cracking, hot cracking, and loss of strength etc. [25,26,27,28]. Furthermore, for non-heat-treated aluminum alloys such as 5A06, the heat-affected zone (HAZ) loss in strength is primarily due to the annealing effect of the cold working microstructure [29]. For heat treatment of 7A05 alloy, the strength loss is usually caused by over-ageing [30]. In particular, the fusion zone of dissimilar welded joints exhibited complicated microstructure composition. It has been reported that the welding quality of dissimilar aluminum alloys is not satisfactory [31,32,33]. Thus, the mechanical property assessment and microscopic study of 7A05-T6/5A06 dissimilar aluminum alloy welded joint should be investigated in detail.

In this paper, microstructural characterization and mechanical properties and of the 7A05-T6/5A06-O dissimilar welded joint of the ring-stiffened closed cylindrical sandwich shell was investigated. The microstructure evolution along the dissimilar joints was discussed. This project is a primary battery cabin shell, which is a one-time use of the product, so this paper does not consider the mechanical properties of welded joints in a corrosive environment. In view of the excellent compressive and compressive stability of the structure, we will consider the long-term use of the structure underwater. In the following projects, we will make a special assessment of the mechanical properties of welded joints under a corrosive environment, and there will be a special paper to discuss.

## 2. Materials and Methods

The structure of the welding specimen is illustrated in Figure 1. The outside shell and inner shell were, respectively, made of 5A06-O aluminum alloy and 7A05-T6 aluminum alloy. The welded joints were welded with butt joint using TIG. Here, the aluminum alloy wire ER5356 with a diameter of 1.6 mm was used as the welding material [34,35]. The details of the material and the wire are shown in Table 1. The weld break was symmetrical to 70°. The weld surface was removed by acetone before welding; then, the polishing machine was used to remove the oxide film of the surface. Finally, the specimens were dried in a drying box. The protective gas used high-purity argon with a purity of 99.999% and a gas flow rate of 24 L/min. Welding current, welding voltage and welding speed were 260 A, 25 V and about 200 mm/min, respectively [34,35]. According to the above process parameters, the workpiece preheating temperature was controlled at 80 ± 5 °C [36]. After the first layer of the bottom of the weld joint was welded, the specimens were cooled to 80 ± 5 °C to complete the weld of the second layer. About three months after the completion of the welding, the tests were conducted. The key process parameters were discovered through experiments. Finally, one of the process parameters was determined to be the best according to the orthogonal test method [37].

**Figure 2:**
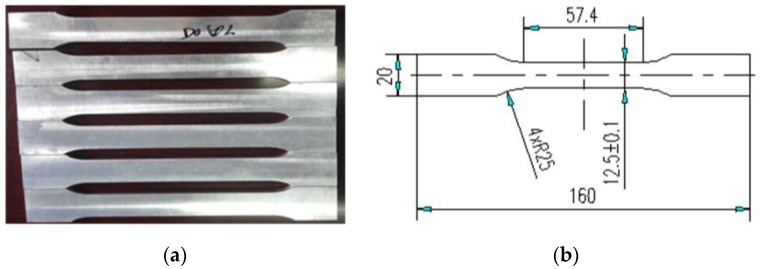
Specimens: (**a**) tensile specimens; (**b**) the dimensions (mm) of the tensile specimens.

**Figure 3:**
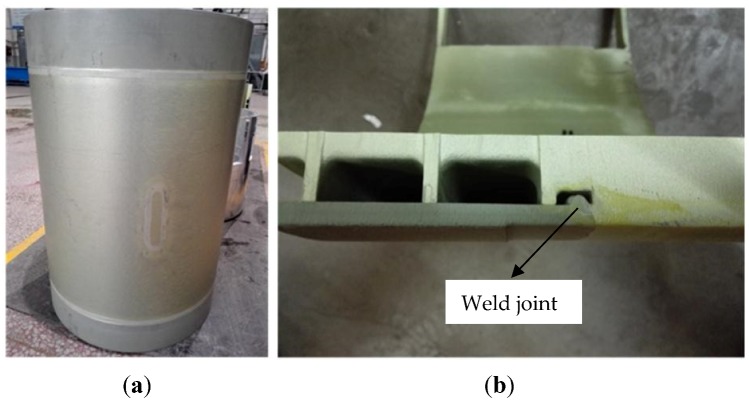
Physic structures of welded sample: (**a**) the 5A06-O/7A05-T6 welded sample; (**b**) the cross section of 5A06-O/7A05-T6 welded joint.

The tensile tests were carried out in accordance with GB/T 16865-2013 [38]. The tensile specimens and the dimensions of the tensile specimens are presented in Figure 2a,b. The tensile test sample was taken from the radial direction of the sample shown in Figure 3 for every 36° and 10 samples were selected. The tensile test was carried out on an MTS-810 tensile tester at a rate of 2 mm/min.

With the VMH-I04 micro hardness tester according to GB/T 4340.1-2009 [39], the test force was 0.9807 N (100 g) and the distance of two points was around 0.85 mm. Vickers hardness gradient test was conducted and the hardness value was given in 308HV0.1.

**Figure 4:**
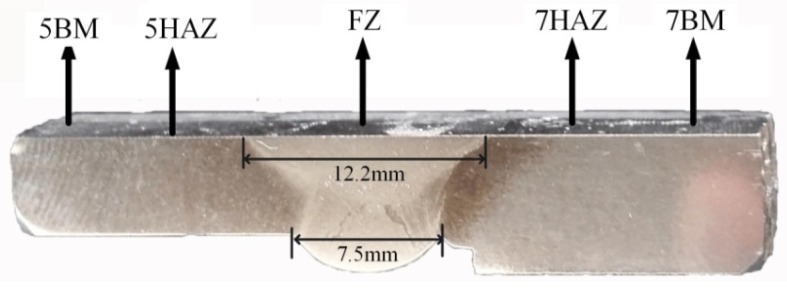
Optical image of the cut-out after electrochemical polishing.

0.5 mm sheets were cut from the block sample shown in Figure 4 and grounded to about 50 mm to produce a sample with φ3. Using 3% perchloric acid alcohol as electrolyte, the perforated film samples were obtained by double jet electrolysis at −20 °C and 75 V. Then the argon ion was thinned for 0.5 h. The microstructures of the distinct regions were observed by transmission electron microscopy (TEM) to obtain the species and distribution of precipitates in this region.

The specimens were ground with emery paper down to 1000 mesh first, and then electropolished by 10% potassium per chlorate alcohol at 30 KV for about 12 s. The grain structure of the weld and fused area was characterized by EBSD (EDAX, Mahwah, NJ, USA). XRD (Rigaku, Akishima, Japan) was used to observe the species of the precipitated joints.

## 3. Results

### 3.1. Mechanical Properties of the Welded Joints

Figure 3a shows the 5A06-O/7A05-T6 test sample of welded shell and a specimen for hardness, and EBSD and XRD measurements were cut from the sample. The section of the welded joints, which were cut from the sample, was presented in Figure 3b. Figure 4 shows the sample after electrochemical polishing. Five different zones are identified, including two base metal (5BM and 7BM), the fusion zone (FZ), and heat-affected zones. The welding is performed by single-sided welding and double-sided forming. Figure 4 shows in the partial cross section of the welded joint. Here, the width of the top surface and the lower surface of the weld are about 12 mm and 7.5 mm, respectively. The back-forming height is approximately 2 mm.

**Figure 5:**
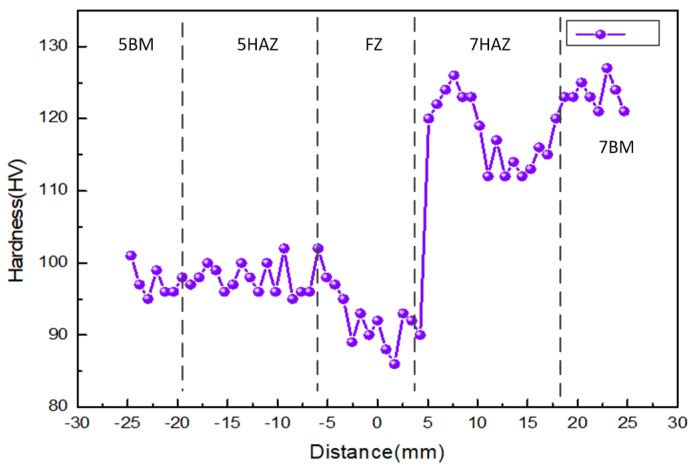
Micro-hardness across the weld.

Hardness distribution of the welded joint is shown in Figure 5. The test surface is the normal direction of the weldment. The zero point is chosen at the center of the welded joint, and the left is 5A06BM and the right is 7A05BM. The fusion zone of the weldment is a little lower than that of the 5A06-O base material; moreover, its average hardness is about 10% lower than that of the 5A06-O base material and the lowest value is 86 HV. The hardness of the heat-affected zone at 5A06 side only changed a little compared to that of 5A06-O base metal. There was not any significant decrease in the hardness of the 7A05 heat-affected zone, which is about 6–11 mm from the weld center. The hardness of the weld zone of 7A05 changed abruptly exhibiting a sudden increase from 90 HV to 120 HV in the length of 1 mm. A softening zone with the minimum hardness of about 110 HV was observed near the center of the welding center 10~18 mm at the 7A05 base material side.

**Figure 6:**
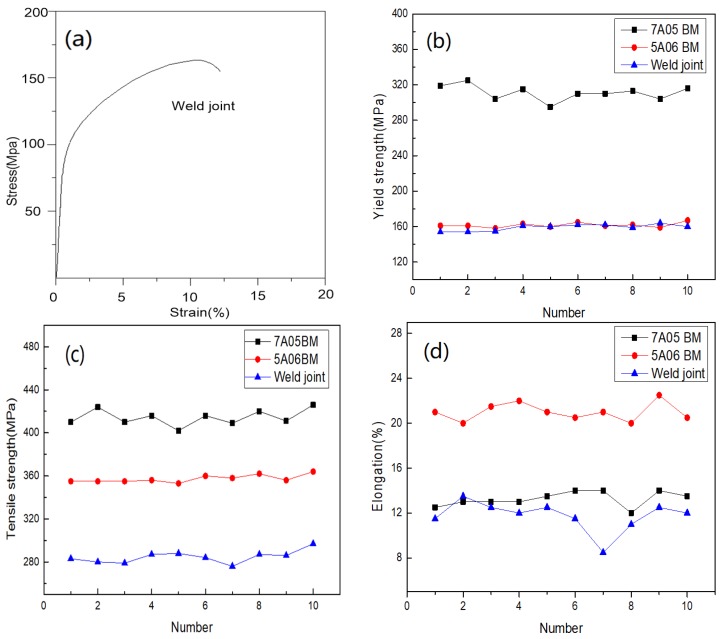
Mechanical properties of the sample: (**a**) tensile stress-strain curves of the sample; (**b**) yield stress of 5A06BM, 7A05BM and joint; (**c**) tensile stress of 5A06BM, 7A05BM and joint; (**d**) elongation of 5A06BM, 7A05BM and joint.

The tensile properties of the welded joint and the base metal are given in Table 2, and tensile stress-strain curve of the welded joint is shown in Figure 6a. As showed in Figure 6b–d, the average tensile strength of the welded joint was 281.08 MPa, which was 78.87% of 5A06-O base material and 67.83% of the base material of 7A05-T6. The average yield strength of the welded joint was very close to the yield strength of the 5A06-O base material, and the average yield strength of the 7A05-T6 base material was quite different. The elongation of the welded joint is 84.29% of the base metal of 7A05-T6 and 54.07% of 5A06-O base material. The welded joint coefficient of the joint reached 78.87% of 5A06-O base metal, which was not lower than that of friction-stir welding [31,32,33]. The elongation and yield strength of the welded joints are ideal [34,35].

**Figure 7:**
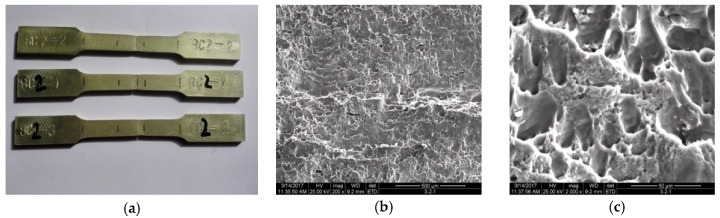
Fracture of the specimens: (**a**) mechanical testing of the weld joints; (**b**) fracture surface of tension specimen; (**c**) fracture surface of tension specimen.

As shown in Figure 6a and Figure 7a, after the deformation of the welded joint, there is a significant necking phenomenon after the occurrence of fracture in the fusion zone when the maximum stress was reached. The fracture surface of the tensile test showed that the dimple of the fracture is obvious without the phenomenon of intergranular fracture as shown in Figure 7b,c.

### 3.2. EBSD Analysis of the Grain Structure

**Figure 8:**
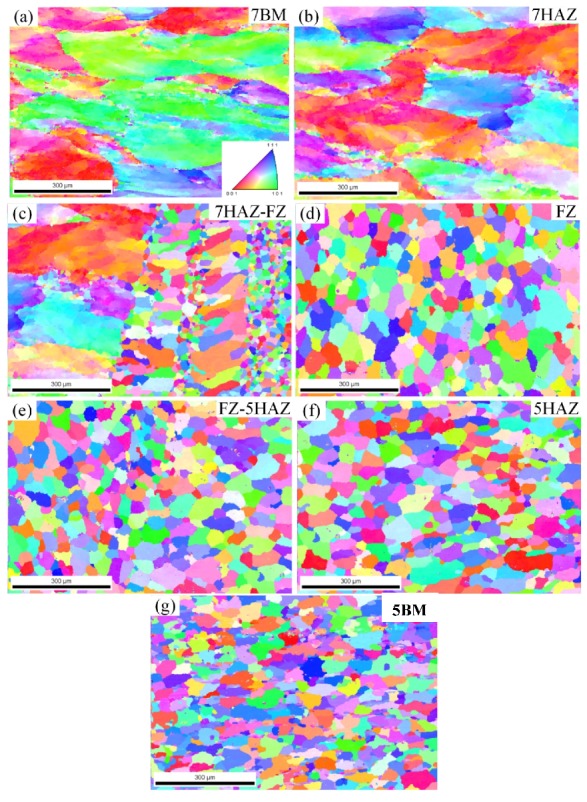
EBSD study of the microstructures of different parts of the two alloys in the weldment. The parts correspond to the EBSD images are indicated in the top right corners. (**a**) 7BM; (**b**) 7HAZ; (**c**) 7HAZ-FZ; (**d**) FZ; (**e**) FZ-5HAZ; (**f**) 5HAZ; (**g**) 5BM.

Figure 8 shows the EBSD images of the seven areas of the welded joint. The direction of observation is the normal direction of the section of the welded joint. The illustration in Figure 8 defines the particle-oriented color coding. The microstructure of the 7A05 base material (see Figure 8a) and the 7A05 heat-affected zone (see Figure 8b) are the distinct fibrous structures. The average particle size of the 7A05HAZ region is about 168 μm, which is equivalent to the average particle diameter (170 μm) of the base material. However, in the heat-affected area the grain larger than 180 μm accounted for about 63%, while the base metal is only about 44%. This indicates that there is a certain growth situation in 7HAZ grain compared to 7BM, which was caused by the heat input when welding. As showed in Figure 8c, the average width of the fused region between 7A05HAZ and welds is about 130 μm, and there is a clear boundary between 7A05HAZ and HZ. Two sub-regions appeared, in which the grain orientation is randomly distributed, and the grain size is not uniform. The grain size near the welding area is about 140 μm, and the grain size of the sub-region is obviously different from that of the other fusion sub-region with the size less than 50 μm. The grain size of the sub-region near the 7A05HAZ is larger than 30 μm and the maximum grain size is about 108 μm. The grains size of the fused sub-regions is smaller than the grain of the sub-regions near the welding zone and the grains are more uniform. The heat input of the weld resulted in the presence of partial recrystallization in this area. As showed in Figure 8d, the grains of the welding zone in the welded joint are fine equiaxed, and the grain orientation in this region is randomly distributed. Most of the grain sizes are distributed in the range of 30–70 μm and the average size is 52 μm. The maximum grain size is about 130 μm, and most of the grains are high angle grain boundaries. As showed in Figure 8e, the fusion zone between the weld and the 5A06 heat-affected zone is not obvious. The area is in an average grain size of about 47 μm, slightly smaller than the average size of the welding area, and larger than for 5A06 base metal (40 μm). As showed in Figure 8f, the EBSD image of the 5A06 heat-affected zone is equiaxed, with an average grain size of about 55 μm, which is larger than that of the base metal. As showed in Figure 8g), the average grain size of the 5A06 base metal is about 40 μm, and the grains are equiaxed.

### 3.3. TEM and XRD Analysis of the Precipitate Structure

**Figure 9:**
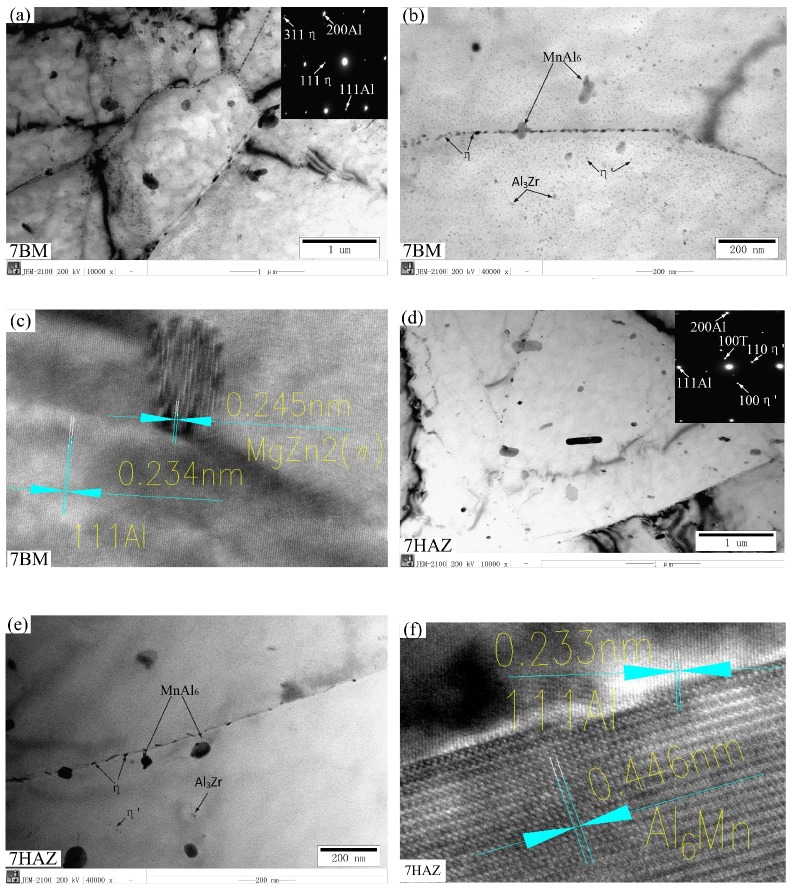

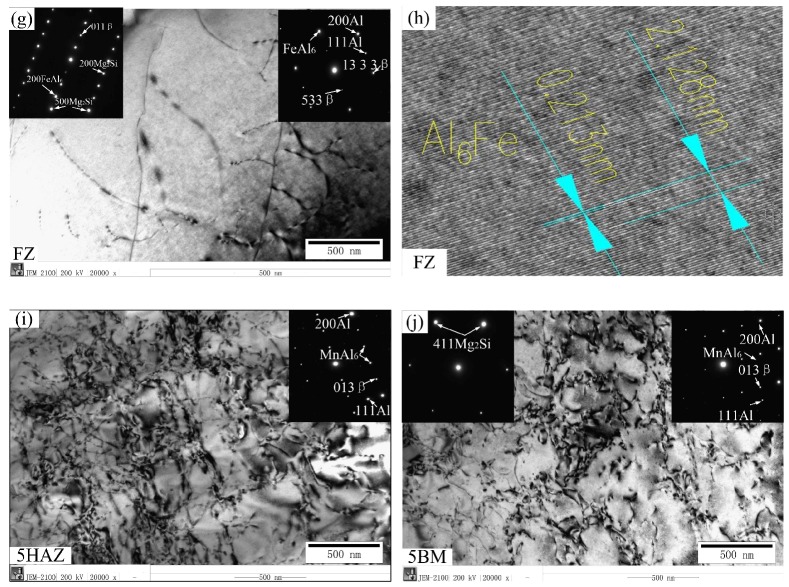
TEM study of the microstructure of different parts of the two alloys in the weldment: (**a–c**) microstructure of the base metal (7BM); (**d–f**) microstructure of the heat-affected zone (7HAZ) adjacent to the base metal; (**g,h**) microstructure of the fusion zone; (**i**) microstructure of the heat-affected zone (5HAZ) adjacent to the base metal; (**j**) microstructure of the base metal (5BM).

Figure 9a–c shows in the microstructure of the base metal of 7A05-T6. The matrix contains high-density dislocations, small size η′ and η precipitated phases, and sub-structures. Horseshoe-shaped Al3Zr particles and many fine dispersion η′ phases are observed. The discontinuous coarse η phase and the formation of precipitation-free zone are precipitated on the grain boundary. Figure 9d–f shows in the microstructure of 7HAZ. The sub-crystal structure is exhibited. The η′ phase of the heat-affected zone is smaller than that of the nanometer granular η′ phase dispersed the base metal; however, the distribution density is obviously reduced. The dislocation density is also reduced in the 7HAZ region. The softening of the 7HAZ region is mainly related to the precipitated phase and the drastic reduction of the dislocation density. Figure 9g,h is the microstructure of the welding zone of the weld. These are the more difficult phase (Mg_2_Si-β) and iron-bearing impurity phase. Compared with 5A06BM and 5A06HAZ, the second strengthening phase is not obvious, and the dislocation density is lower. This is one reason the strength and hardness of the area are the weakest of the welded joints. As shown in Figure 9i, the 5A06 heat-affected zone is similar to the base metal structure and has a higher dislocation density. Figure 9j is the microstructure of 5A06BM, and more hardening phases (Al_3_Mg_2_-β) and some insoluble phases (Mg_2_Si) are precipitated. From the TEM image, the base metation spot, the XRD l region contains more manganese phase and a small amount magnesium silicon phase. After diffraction, we confirmed that it is MnAl_6_ and Mg_2_Si. Matrix metal contains high-density dislocations.

**Figure 10:**
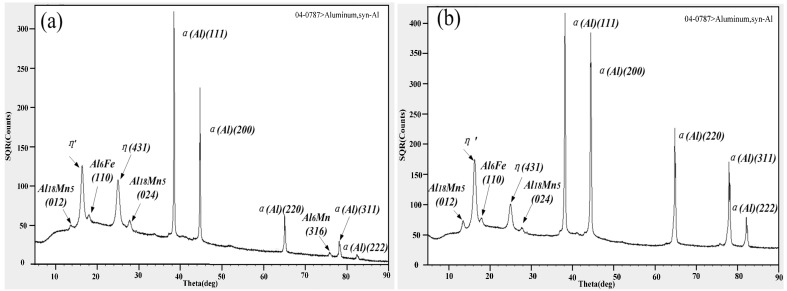

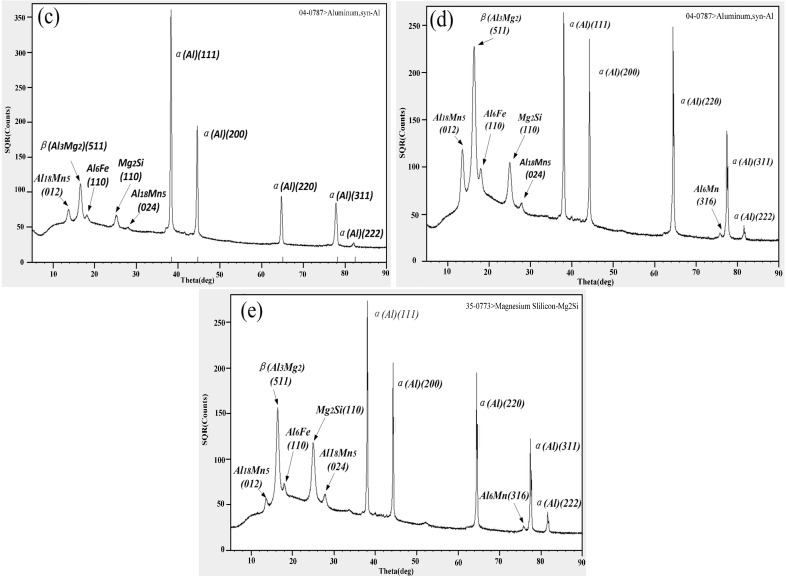
XRD study of the microstructure of different parts of the two alloys in the weld joint: (**a**) microstructure of the base metal (7BM); (**b**) microstructure of the heat-affected zone adjacent to the base metal (7HAZ); (**c**) microstructure of the fusion zone; (**d**) microstructure of the heat-affected zone adjacent to the base metal (5HAZ); (**e**) microstructure of the base metal (5BM).

The XRD test of the different parts of the welded joint is illustrated in Figure 10. According to Figure 10a,b, 7A05 base material and heat-affected zone contains some Mn-containing phase, and a small amount of Fe-containing phase. There are more η′ and η phases. As shown in Figure 10c–e, the strengthening phase (Al_3_Mg_2_-β) precipitated in the weld zone is much less than that of the 5A06 base metal and the 5A06HAZ region, which is consistent with the TEM results. The three regions all contain a certain iron-containing impurity phase and a poorly soluble magnesium silicon phase.

## 4. Discussion

In the plate butt welding, owing to air circulation, the temperature around the weld heat diffused quickly. It takes about 1 h for the weld to cool to room temperature. In this experiment, due to the particularity of the structure, as shown in Figure 1, the closed cavity formed at the bottom of the weld, outer shell and inner shell is not conducive to the diffusion of heat. Therefore, about 8 h after welding, the temperature can reach room temperature, which is equivalent to an 8 h quenching process. This process takes such grain coarsening of the weld and the gradual dissolution of the strengthening phase, resulting in loss of weld strength.

**Figure 11:**
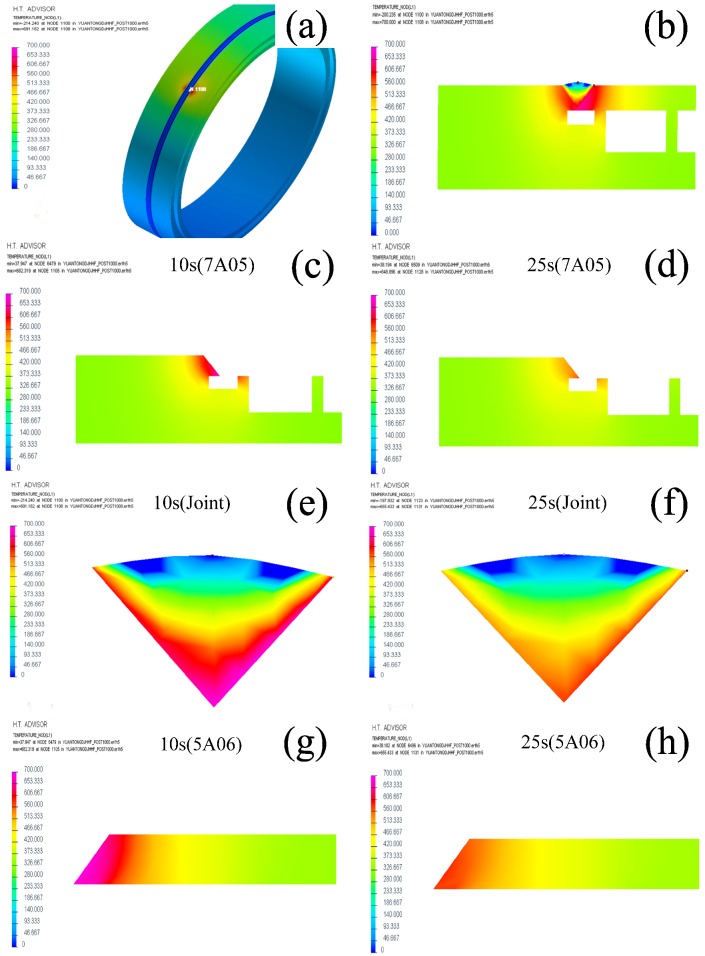
Temperature field distributions: (**a**) simulation of temperature field distribution of welded joints; (**b**) temperature distribution of welded joint profiles; (**c**–**d**) 7A05BM profile temperature field distribution; (**d**,**e**) weld profile temperature field distribution; (**g**,**h**) 5A06BM profile temperature field distribution.

The evolution of microstructures is mainly associated with the heat input during welding, especially the maximum temperature. Finite element software has been used to simulate the temperature field and stress field in the welding process. The welding process of the welding joint is simulated by SYSWELD (version2015, ESI Group, Paris, France) in this paper, which is professional software for welding simulation. The temperature field around the weld and the temperature of each area of the welded joint is obtained. For the simulation, a double ellipsoidal heat source was expected to be adopted and used at the fusion zone and its vicinity [40]. The total number of unit cells was 20,100 and the number of mesh nodes is 24,800. The color scales indicate temperatures in Celsius. The maximum temperature that can be reached during the welding is plotted in Figure 11 against the distance from the weld center. The welding rate and the heat input are 200 mm/min and 1950 J/mm. The temperature of the weld center is a little higher. The high temperature region is vast and the high temperature range of the 7A05 side is relatively small. This situation may arise because the 7A05 size has thicker, faster heat dissipation. In addition, the bottom of the weld and the right side of the closed cavity also have a greater impact on heat dissipation, resulting in a larger high-temperature area of the 5A06 side and longer duration.

Since the chemical composition of 7A05 and 7N01 (Al base, 4.8 wt % Zn, 1.3 wt % Mg) is analogous, it has similar melting and recrystallization temperatures. The melting point and recrystallization temperature of 7A05 is 607–643 °C and 460–500 °C, respectively [41]. This low-magnesium 7XXX aluminum alloy has the following precipitation sequence: GP zone′ phase (disc) → η phase (rod) [42,43]. The temperature range of nucleation and growth of the precipitates is as follows: GP zone is 20–120 °C, η′ is 120–250 °C and η is 150–300 °C [44,45]. It can be seen from Figure 11(b,c,e) that the maximum temperature of the 7HAZ near the FZ is above 600 °C during the welding process, indicating that the recrystallization process has been carried out here, consistent with the results in Figure 8c. From Figure 9b–e, the heat input during the welding process results in the dissolution of the η and η′ precipitates, leading to a loss of strength of 7HAZ and a softening zone. 7HAZ heat-affected zone along the heat direction is followed by quenching and the over-aging phenomenon. Therefore, it can be divided into hot and quenching area and over-aging area. The original precipitated phase in the quenching zone is solid-dissolved into the aluminum matrix to form a supersaturated solid solution. After a period, the solid solution precipitates the η′ (MgZn_2_) phase, resulting in natural aging strengthening, resulting in a higher hardness. These results match with the hardness curve indicated in Figure 5. From the center of the weld 10–18 mm range, the hardness decreased with the minimum value of about 110 HV. This reason is that the averaging zone is far away from the weld zone, and the aging temperature above the 7A05 aluminum alloy is lower than the solution treatment temperature, which caused the η′ phase to grow and coarsen. The decreases of cooling speed leads to the further dissolution of the strengthened phase in this region. The grain of 7HAZ larger than 180 μm occupies 63% in Figure 8b, and the grain of 7BM larger than 180 μm accounts for about 44% in Figure 8a, which shows that 7HAZ has a certain growth condition agreeing with the analysis results. However, the maximum particle size of 7HAZ is about 260 μm, and the maximum particle size of 7BM is about 320 μm. It is shown that the grain distribution in 7HAZ is more uniform than that in 7BM, which may be related to the longer quenching time after welding.

It can be seen from Figure 11b,e,g that the 5HAZ-FZ zone temperature range is approximately 370–700 °C, the 5HAZ-5BM zone temperature range is approximately 160–370 °C, and the 5BM temperature is about 160 °C. Since the chemical composition of 5A06 is similar to AA5083, it has similar melting and recrystallization temperatures. Melting point of 5A06 is 570–640 °C and the recrystallization temperature is 343–371 °C [46]. It is generally thought that β-phase precipitates in binary Al-Mg alloys are formed following the reaction [47,48,49]:

Solid solutionα → GP zones → β″ → β′ → β

GP zones stand for Guinier-Preston zones: β″ is an L12 ordered phase (composition Al_3_Mg), β′ is a semi-coherent hexagonal intermediate phase and β is the equilibrium phase having a complex FCC structure In Al-Mg alloy aged between 100 and 250 °C, the β′-phase forms first around 100 °C and β-phase appears around 200 °C in the matrix of the nearly complete depletion of Mg [50]. However, it was observed that β-phase precipitates below 200 °C [51].

5HAZ occurred in the recrystallization phenomenon in the welding process. The grain has increased significantly and coincides with the results of EBSD (see Figure 8e,f). From the XRD analysis of Figure 10d,e, the precipitation strengthening of 5HAZ is much greater than that of 5BM. The enhancement of the strengthening phase may counteract the weakening effect of strength and hardness thanks to coarse grain. Therefore, the hardness of 5HAZ related to that of 5BM has a very small change. 

## 5. Conclusions

In this paper, TIG and single-sided welding and double-sided forming have been used to weld the 7A05-T6/5A06-O heterogeneous aluminum alloy circular-welded joint of a ring-stiffened closed cylindrical sandwich shell, and the welding performance is evaluated. The main results can be summed up as follows:

1. The tensile strength and yield strength of the welded joint reached 78.87% and 97.24% of the 5A06 base metal, respectively, and the elongation reached 84.29% of the 7A05-T6 base metal. Its mechanical properties after external hydraulic test and lifting test were verified to meet engineering requirements.
2. The main reason for the weakest area of the weld zone is that the grain is coarsened in the region. The enhanced amount of precipitates is less or the strengthening phase dissolves during long periods of quenching, moreover, there are more iron-containing impurity phases.
3. The hardness curve of the welded joint is asymmetrical. The width of the weld is approximately 12 mm, and the hardness of the weld center is the lowest (about 86 HV). 7HAZ is divided into the quench zone and over-aging zone. The original precipitated phase of the quenching zone is dissolved in the aluminum matrix to form a supersaturated solid solution. After a period, the solid solution will precipitate η′ (MgZn_2_) phase, resulting in natural aging strengthening, and achieving a higher hardness. The hardness reduction occurred in the distance from the center of the weld 10–18 mm range, which is the over-aging area with a minimum value of about 110 HV. The reason is that over-aging area is far from the weld, and the temperature is higher than the aging temperature. 7A05, however, is lower than the solution treatment temperature of 7A05, resulting in η′ phase aggregation growing and coarsening. Moreover, η′ (MgZn_2_) enhanced phase dissolved fully and the dislocation density decreased rapidly, resulting in softening of the over-aging area due to the long quenching effect of 7HAZ area. However, the hardness of the softening area is higher than the weld zone, and the weld zone is still the weakest position of the welded joint.

## Figures and Tables

**Table 1:** Chemical composition (wt. %) of the 7A05 and 5A06 base metal and ER5356 welding wire.

Base Metal	Si	Fe	Mg	Mn	Cr	Zn	Cu	Zr	Ti	Al
7A05	0.077	0.15	1.37	0.22	0.09	4.58	0.0078	0.15	0.044	Balance
5A06	0.12	0.13	6.2	0.62	-----	0.232	0.11	-----	0.034	Balance
ER5356	0.037	0.13	4.59	0.12	0.11	0.007	0.001	-----	0.1	Balance

**Table 2:** Mechanical properties of the 7A05BM, 5A06BM and weld joint.

Base Metal	Yield Strength (MPa)	Tensile Strength (MPa)	Rupture Strain (%)
7A05-T6	414.4	311.6	13.56
5A06-O	356.4	161.8	21.14
7A05/5A06	281.08	157.33	11.43
